# Oligosaccharides increase the genotoxic effect of colibactin produced by *pks+ Escherichia coli* strains

**DOI:** 10.1186/s12885-021-07876-8

**Published:** 2021-02-17

**Authors:** Manon Oliero, Annie Calvé, Gabriela Fragoso, Thibault Cuisiniere, Roy Hajjar, Ulrich Dobrindt, Manuela M. Santos

**Affiliations:** 1 Nutrition and Microbiome Laboratory, Institut du cancer de Montréal, Centre de recherche du Centre hospitalier de l’Université de Montréal (CRCHUM), 900 Rue Saint Denis, Montreal, QC H2X 0A9 Canada; 2 Department of Medicine, Faculty of Medicine, Université de Montréal, 2900 boulevard Édouard-Montpetit, Montréal, QC H3T 1J4 Canada; 3 Department of Surgery, Faculty of Medicine, Université de Montréal, 2900 boulevard Édouard-Montpetit, Montréal, QC H3T 1J4 Canada; 4 Institute of Hygiene, Section Microbial Genome Plasticity, University of Münster, Mendelstraße 7, 48149 Münster, Germany

**Keywords:** *E. coli*, Colorectal cancer, Colibactin, Genotoxin, Inulin, Galacto-oligosaccharides, Iron

## Abstract

**Background:**

Colibactin is a genotoxin that induces DNA double-strand breaks that may lead to carcinogenesis and is produced by *Escherichia coli* strains harboring the *pks* island. Human and animal studies have shown that colibactin-producing gut bacteria promote carcinogenesis and enhance the progression of colorectal cancer through cellular senescence and chromosomal abnormalities. In this study, we investigated the impact of prebiotics on the genotoxicity of colibactin-producing *E. coli* strains Nissle 1917 and NC101.

**Methods:**

Bacteria were grown in medium supplemented with 20, 30 and 40 mg/mL of prebiotics inulin or galacto-oligosaccharide, and with or without 5 μM, 25 μM and 125 μM of ferrous sulfate. Colibactin expression was assessed by luciferase reporter assay for the *clbA* gene, essential for colibactin production, in *E. coli* Nissle 1917 and by RT-PCR in *E. coli* NC101. The human epithelial colorectal adenocarcinoma cell line, Caco-2, was used to assess colibactin-induced megalocytosis by methylene blue binding assay and genotoxicity by γ-H2AX immunofluorescence analysis.

**Results:**

Inulin and galacto-oligosaccharide enhanced the expression of *clbA* in *pks+ E. coli*. However, the addition of 125 μM of ferrous sulfate inhibited the expression of *clbA* triggered by oligosaccharides. In the presence of either oligosaccharide, *E. coli* NC101 increased dysplasia and DNA double-strand breaks in Caco-2 cells compared to untreated cells.

**Conclusion:**

Our results suggest that, in vitro, prebiotic oligosaccharides exacerbate DNA damage induced by colibactin-producing bacteria. Further studies are necessary to establish whether oligosaccharide supplementation may lead to increased colorectal tumorigenesis in animal models colonized with *pks+ E. coli*.

**Supplementary Information:**

The online version contains supplementary material available at 10.1186/s12885-021-07876-8.

## Background

Colorectal cancer (CRC), the 3rd most prevalent cancer worldwide, is caused by various factors such as genetics, diet, environment, lifestyle and the gut microbiome [1]. CRC and colitis-associated CRC patients display an unbalanced gut microbiome, which leads to significant differences in species richness and diversity compared to healthy individuals [2]. For instance, the proportion of beneficial bacteria such as *Bifidobacterium,* *Clostridiales* and *Faecalibacterium* are decreased while the relative abundance of potentially harmful bacteria belonging to the *Enterobacteriaceae* family, such as some *Escherichia coli* strains, is higher [3].

*E. coli* that harbor the polyketide synthase (*pks)* island can be part of the microbial pool colonizing the gut of patients with inflammatory bowel disease [4], patients with familial adenomatous polyposis [5] or CRC, as well as healthy individuals [6]. This genomic island encodes the components of a polyketide/non-ribosomal peptide hybrid biosynthesis pathway that is responsible for the expression of the genotoxin colibactin [7]. Colibactin causes DNA double-strand breaks (DSBs) in mammalian cells and leads to cell cycle arrest, senescence and chromosomal abnormalities [8, 9]. More recently, a distinct mutational profile has been identified in CRC, suggesting a direct mutational process resulting from past exposure to colibactin-producing bacteria [10]. Furthermore, mono-colonization of *pks+ E. coli* in murine models showed a direct link between colibactin production and colon carcinogenesis [4, 5]. Importantly, about 20–22% of healthy individuals are colonized by *pks*+ *E. coli* [5, 6], and these individuals may be at higher risk of developing CRC.

Since complete eradication of *pks+ E. coli* from the gut microbiome is not feasible to reduce CRC risk [11–13], we aimed at regulating the genotoxin by using prebiotics, a major regulator of the gut microbiota metabolism [14]. Prebiotics are fermentable fibers, which include oligosaccharides, that have beneficial effects on intestinal health through the maintenance of mucosal integrity, and most importantly, through the promotion of beneficial bacteria feeding on prebiotics to generate short chain fatty acids [15, 16]. For example, inulin used in combination with a probiotic decreased the viability and growth of *E. coli* [17, 18], whereas galacto-oligosaccharides (GOS) was shown to reduce the adhesion of enteropathogenic *E. coli* to cultured cells [19]. However, little is known about the direct effect of prebiotics supplementation on genotoxin expression from *pks+ E. coli* present in the gut microbiota.

In this study, we investigated the effects of inulin and GOS, two oligosaccharides known to regulate bacterial metabolism, on colibactin using two *pks+ E. coli* strains. The effect of supplementation with iron, a known colibactin regulator [20, 21], was additionally assessed. Finally, the effects of inulin and GOS on colibactin-related genotoxicity were evaluated using the adenocarcinoma cell line Caco-2.

## Methods

### Reagents

Inulin was purchased from Sigma Aldrich (Missouri, USA) and GOS from Carbosynth (Compton, UK), and were prepared as stock solutions of 80 mg/mL. Ferrous sulfate (FeSO_4_) was purchased from Sigma Aldrich and 100 mM stock solutions were prepared.

### Bacterial strains and growth conditions

*E. coli* strains used in this study: control strain *pks*-* E. coli* K-12, which is colibactin-negative (ER2738, New England BioLabs, New York, United States); the murine *pks+ E. coli* NC101 strain (a gift from Dr. Christian Jobin, Cancer Microbiota & Host Response, UF Health Cancer Center, University of Florida); and the engineered *E. coli* Nissle 1917 (EcN) strains carrying a chromosomal translational fusion consisting of the promoterless *luxABCDE* construct and the promoter of one of the four genes *clbA*, *clbB*, *clbQ* or *clbR* [7]. Frozen bacterial glycerol stocks were grown in lysogeny broth (LB) (Wisent Inc., Québec, Canada). For experiments, bacteria were grown at 37 °C, shaking at 150 rpm in standard minimal medium (M9). For infection of Caco-2 cells, bacteria were grown in Eagle’s Minimum Essential Medium (EMEM) (Wisent Inc) at 37 °C at 150 rpm.

### Cell culture and in vitro infection

The human colonic adenocarcinoma cell line Caco-2 (ATCC® HTB-37™) was a gift from Dr. Petronela Ancuta, (Department of Microbiology, Infectiology and Immunology, Université de Montréal). Short Tandem Repeat (STR) analysis for Human Cell Line Authentication was performed using the GenePrint**®** 10 system (Promega, WI, USA) at Genome Québec, Canada. Cells were monitored for mycoplasma contamination using the ABC PCR mycoplasma detection kit (Applied Biological Materials Inc., BC, Canada). Cells were grown in EMEM supplemented with 20% fetal bovine serum (FBS) (Thermo Fisher). Cells were maintained in 75 cm^2^ culture flasks at 37 °C in a 5% CO_2_ (v/v) incubator in a humidified atmosphere.

### Growth curves and luciferase measurements

*E. coli* strains from glycerol stocks were grown in LB at 37 °C at 150 rpm overnight and then sub-cultured at 1/100 dilution in M9 medium. For growth experiments, 1 × 10^7^/100 μl of bacterial cells were inoculated in a transparent 96 well plate (Sarstedt, Nümbrecht, Germany) and were grown with shaking at 37 °C. Bacterial growth (OD_600nm_) was assessed at every hour. For luminescence measurements, 1 × 10^7^/100 μl of bacterial cells were inoculated in a white 96 well plate (Greiner Bio-One, Kremsmünster, Austria) and were grown with shaking at 37 °C. Light emission (luminescence (count/s) expressed as relative light units (RLU)) was recorded every hour in parallel with OD in a Spark® multimode microplate reader (TECAN, Québec, Canada).

### RNA extraction and reverse transcription polymerase chain reaction (PCR)

Total RNA from *E. coli* NC101 grown in LB for 7 h was isolated, and contaminating DNA was removed using DNase I (Biobasic, Ontario, Canada) for 30 min at 37 °C, followed by RNA extraction using the Total RNA Mini-Preps Kit (Biobasic). Reverse transcription PCR was performed on cDNA reverse transcribed from 50 ng RNA using the High Capacity cDNA Reverse Transcription Kit (Thermo Fisher). Real time PCR was performed using the enzyme PowerUp™ SYBR™ Green Master Mix (Thermo Fisher) using the RG 3000A R (Qiagen, Québec, Canada). Primers used are presented in Additional Table 1. Relative quantitation was performed using standard curves constructed from serial dilutions of PCR products [22]. mRNA expression for each gene was determined by direct comparison with the standard curve of the specific target generated in each PCR run. Expression levels of *clbA*, *clbB*, *clbQ* and *clbR* were normalized to 16S rRNA.

### Megalocytosis assay

Quantification of the colibactin-associated genotoxic effect by megalocytosis assay was performed as previously described [23]. Briefly, *E. coli* NC101 and K-12 strains from glycerol stocks were grown in LB at 37 °C, shaking overnight. Strains were sub-cultured in EMEM for 4 h. Caco-2 cells were dispensed (1 × 10^5^ cells/well) in a 24 well tissue culture plate (Falcon) at 37 °C in a 5% CO_2_ atmosphere. After 24 h, Caco-2 cells were infected at a multiplicity of infection (MOI) of 50 with indicated *E. coli* strains. After 4 h of infection, the cells were washed at least three times with phosphate buffer saline (PBS) (Wisent Inc) and incubated for 72 h in cell culture medium supplemented with 200 μg/ml gentamicin (VWR). Cells were fixed with 4% paraformaldehyde (Thermo Fisher) for 15 min, washed and stained with 1 mM methylene blue (Sigma Aldrich). Pictures were taken under a Nikon Eclipse TE300 microscope (Nikon Healthcare, Québec, Canada) and images were acquired using the NIS-Elements BR4.00.03 software (200X magnification). Methylene blue extraction solution was used to quantify cell damage and megalocytosis at 660 nm absorbance (Spark® multimode microplate reader).

### Cell viability assay

Viability of Caco-2 cells was examined using a 3-(4,5-DimethylthiaZA-2-yl)-2,5-diphenyltetrazolium bromide (MTT) (Sigma Aldrich) as previously described [24]. Briefly, after infection the cells were washed with PBS and incubated for 72 h in cell culture medium supplemented with 200 μg/ml gentamicin. Cells were incubated with 10 µL of MTT for 3 h at 37 °C in a 5% CO_2_ atmosphere. After the incubation period, 100 μL of acid-isopropanol (0.04 N HCi in isopropanol) were added and mixed thoroughly to dissolve the formazan crystals. Cell viability was quantified at 540 nm absorbance using the Spark® multimode microplate reader.

### Fluorescent immunostaining of γ-H2AX by In Cell Western assay

Quantification of DNA DSBs was performed using the In Cell Western assay as described [25]. Caco-2 cells were dispensed (1 × 10^5^ cells/well) in a black 96 well plate (Greiner Bio-One) and incubated at 37 °C in 5% CO_2_ atmosphere. After 24 h, Caco-2 cells were infected at MOI 50 with *E. coli* strains. After 4 h of infection, the cells were washed three times with PBS and incubated for 3 h in cell culture medium supplemented with 200 μg/ml gentamicin. Cells were fixed (4% paraformaldehyde), permeabilized, blocked and then incubated overnight with rabbit monoclonal anti-γ-H2AX (BioLabs) at 1/200 dilution. Secondary antibody IRDye™800CW goat anti-rabbit (Biotium, Wisconsin, United States) was applied simultaneously with 1/500 dilution of RedDot™2 (Biotium) for DNA labeling. The DNA and γ-H2AX were visualized using Odyssey® infrared imaging scanner (LI-COR model 9120, Québec, Canada) with red denoting RedDot™2 and green for IRDye™800CW goat anti-rabbit. Images were processed using Image Studio Ver3.1 software.

### Statistics

Experiments were performed at least three separate times and with each condition in triplicate. Results are presented as mean ± SEM. Graphs were drawn using GraphPad Prism (Version 5.0) software, and ANOVA with post-hoc Tukey’s test was used to determine statistically significant results.

## Results

### Inulin and GOS enhance *clbA* expression in EcN

**Fig. 1:**
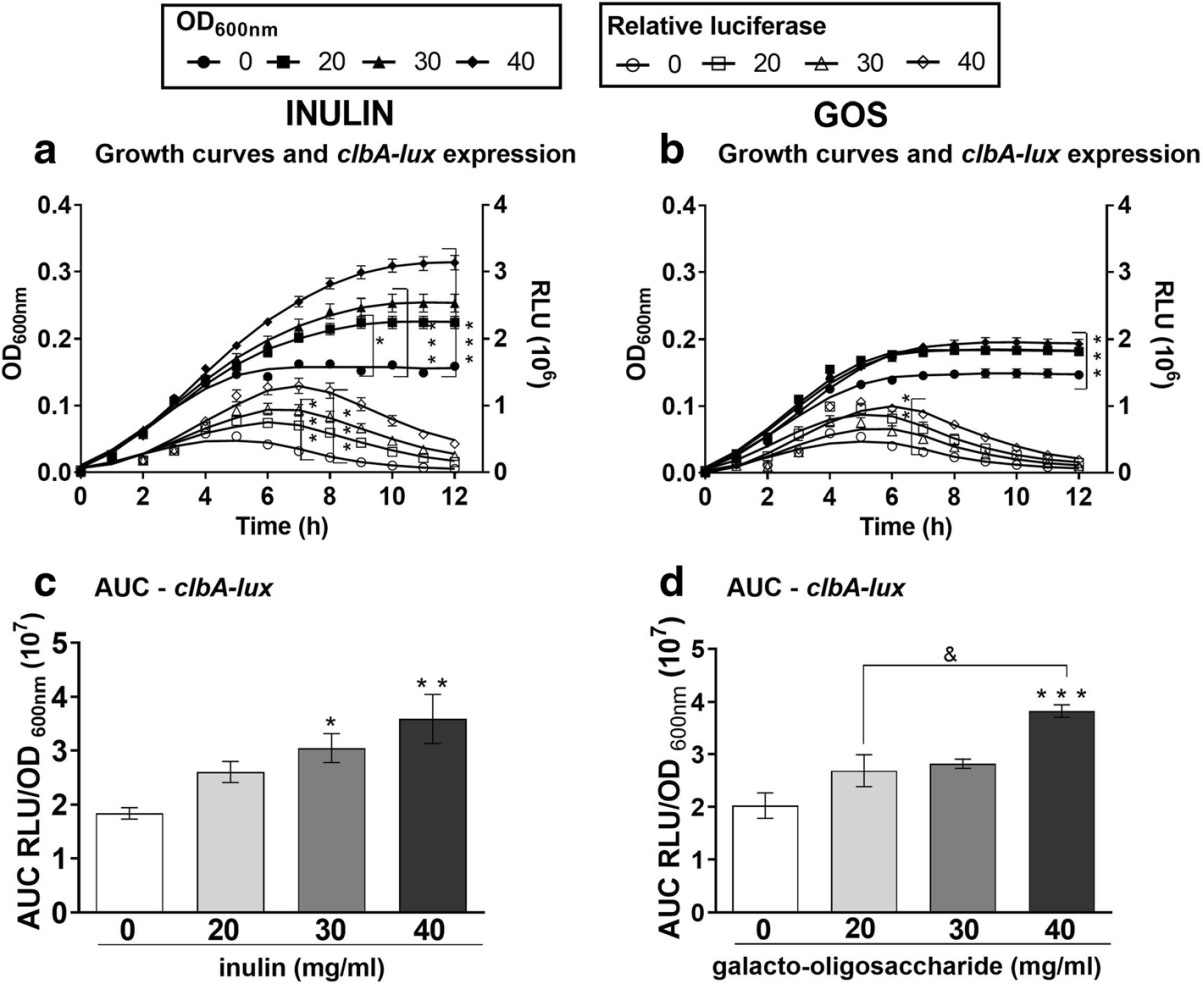
Inulin and GOS increase the *clbA* promoter activity. **a**-**b** Growth curves and luminescence measurement of EcN carrying the translational *clbA*-luciferase reporter fusion. The effect of 0, 20, 30 and 40 mg/mL of inulin (**a**) and GOS (**b**) on growth (OD_600_; filled symbols) and on *clbA*-*lux* relative luminescence (RLU 10^6^; open symbols). **c**-**d** Area under the curve (AUC) of RLU/OD_600_ for inulin (**c**) and GOS (**d**). *P < 0.05, **P < 0.01, ***P < 0.001 compared to control (0 mg/mL oligosaccharides) and &*P* < 0.05; ANOVA

To study the impact of oligosaccharides on the growth of EcN and colibactin expression, we cultured the bacteria in the presence of inulin or GOS. As shown in Fig. 1 a and Supplementary Fig. 1A-C the addition of inulin from 20 mg/mL to 40 mg/mL to minimal media significantly increased bacterial growth (OD_600_). Similarly, GOS supplementation stimulated the growth of EcN, albeit to a lesser extent than inulin (OD_600_, Fig. 1b and Additional Fig. 1D-F).

To determine colibactin expression, the transcript levels of the genes *clbA, clbB, clbQ*, and *clbR* fused to a promoterless luciferase reporter construct (*lux*) were quantified by relative luminescence (RLU, Fig. 1a-b and Additional Fig. 1a-f). As previously reported [7], the *clbA* gene had the highest expression, followed by *clbB*, *clbR* and *clbQ*. For *clbA*, we found an increase in RLU over time, peaking around 7 h. When bacteria were supplemented with 40 mg/mL of oligosaccharides, a significant increase in RLU was seen when compared to control conditions of bacteria grown in the absence of prebiotics (Fig. 1a-b and Additional Fig. 1 A-F).

The area under the curve (AUC), calculated by dividing the RLU by OD_600_, revealed that inulin supplementation increased the expression of *clbA* (Fig. 1c), as well as *clbB*, *clbQ* and *clbR* (Additional Fig. 1G-I), in a dose-dependent manner. In turn, GOS enhanced the expression of *clbA* only at the highest concentration, i.e. 40 mg/mL (Fig. 1d), without affecting the levels of *clbB*, *clbQ* and *clbR* (Additional Fig. 1 J-L).

These results indicate that inulin stimulates the expression of several *clb* genes in EcN, while GOS affects *clbA* expression only.

### Iron decreases the *clbA* expression of EcN stimulated by oligosaccharides

**Fig. 2:**
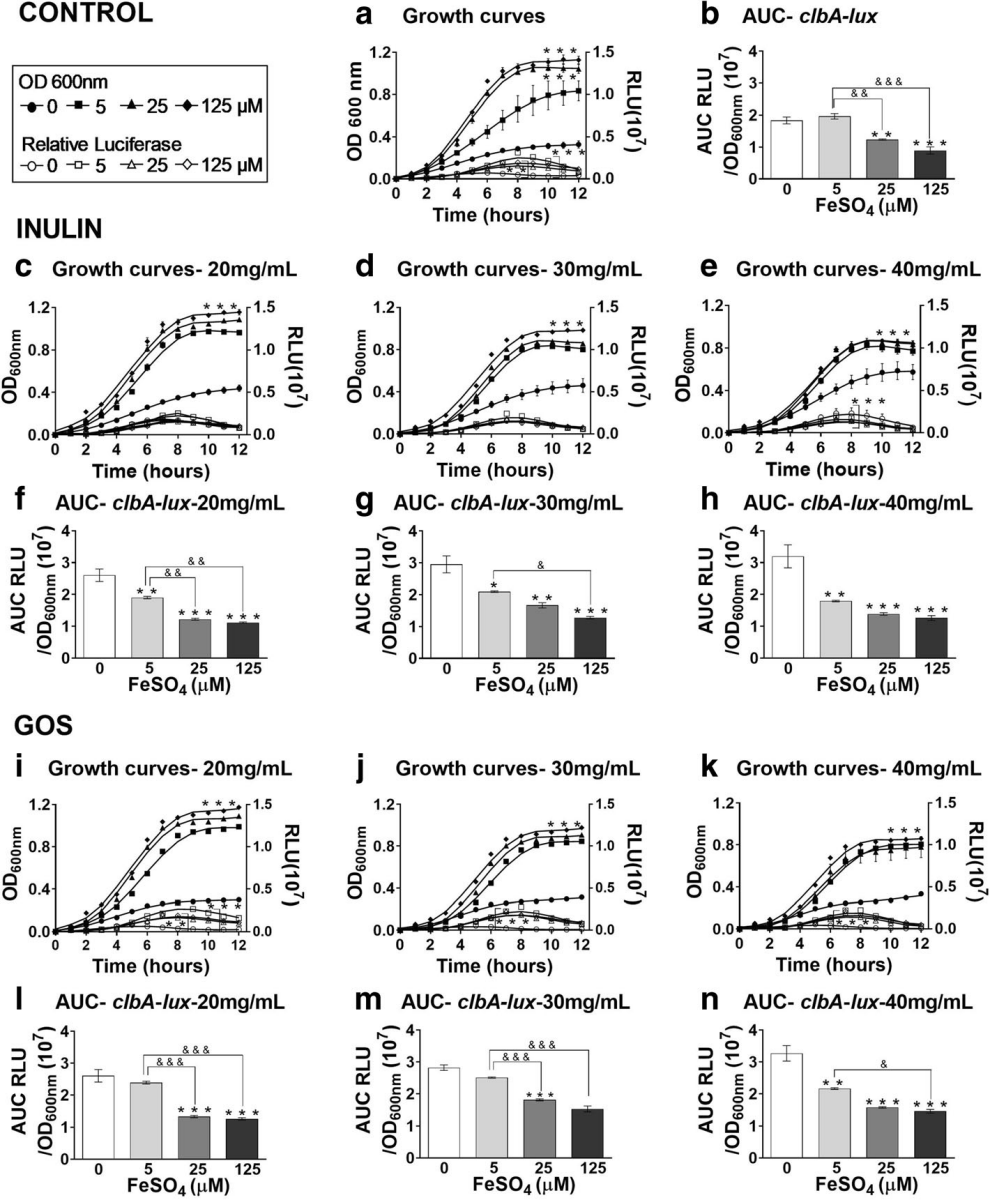
Iron decreases the transcription of *clbA* promoter activity promoted by oligosaccharides. **a** Growth curves and luminescence measurement of EcN the translational *clbA* luciferase reporter fusion and **b** area under the curve (AUC) of RLU/OD_600_, in media containing 5 μM, 25 μM and 125 μM ferrous sulfate without inulin and with **c** 20, **d** 30, **e** 40 mg/mL inulin and **f-h** corresponding area under the curve (AUC) of RLU/OD_600_. Growth curves and luminescence measurement of EcN in media containing 5 μM, 25 μM and 125 μM ferrous sulfate supplemented with **i** 20, **j** 30, **k** 40 mg/mL GOS and **l-n** corresponding area under the curve (AUC) of RLU/OD_600_. *P < 0.05, **P < 0.01, ***P < 0.001 compared to control (0 mg/mL oligosaccharides); &*P* < 0.05, &&*P* < 0.01, and &&&*P* < 0.001; ANOVA

In addition to colibactin synthesis, the *clbA* gene from the *pks* island is also involved in the synthesis of siderophores, such as enterobactin and yersiniabactin, which are small molecules synthesized by bacteria that scavenge and solubilize ferric iron (Fe^3+^) [26]. Iron was previously shown to downregulate the expression of the colibactin gene cluster including the *clbA* gene [21, 26, 27]. Hence, we tested, using the *clbA* reporter construct, whether the addition of iron abrogated the increased expression of the colibactin gene cluster induced by oligosaccharides supplementation. The addition of ferrous sulfate to the minimal medium resulted in significantly increased growth of EcN (Fig. 2a), whereas *clbA* transcript levels were reduced in a concentration-dependent-manner (Fig. 2b), as expected [21, 27]. Addition of inulin increased the growth of EcN as confirmed by our previous experiments (Fig. 2c-e; line with circles). However, when comparing the growth curves of bacteria exposed to increasing inulin concentrations in medium containing ferrous sulfate at 125 μM, the inulin-induced growth was visibly inhibited, as shown in Fig. 2c-e (line with diamonds).

We then calculated the individual luciferase activity levels (AUC of RLU/OD_600_) when the bacteria were grown in medium supplemented with inulin and iron. The addition of 5 to 125 μM of ferrous sulfate to the medium with inulin led to a decrease in *clbA* expression in a concentration-dependent manner (Fig. 2f-h). Similar results using GOS supplementation were obtained regarding bacterial growth (Fig. 2i-k) and *clbA* expression (Fig. 2l-n).

These results indicate that iron supplementation inhibits the effects of oligosaccharides on bacterial growth and *clbA* induction as an indicator for the expression of the colibactin gene cluster.

### Inulin and GOS enhance *clbA* expression in tumor-promoting *E. coli* strain NC101

**Fig. 3:**
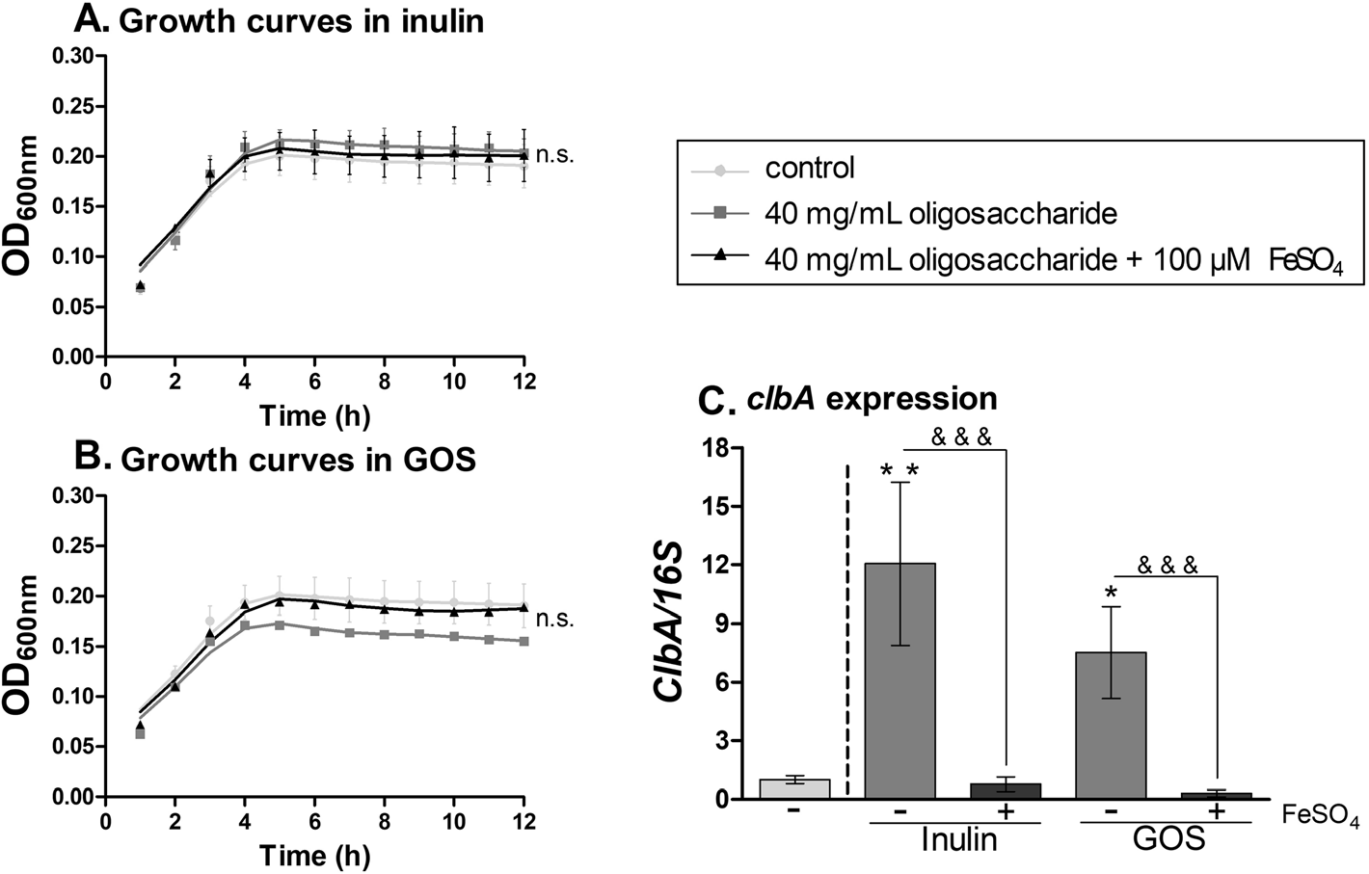
Inulin and GOS increase the expression of *clbA* in *E. coli* strain NC101. **a**-**b** Growth curves of *E. coli* NC101 grown in 100 μM of iron sulfate and 40 mg/mL of inulin (**a**) or 40 mg/mL of GOS (**b**). **c** Fold change of *clbA/16S* rRNA expression in minimal medium supplemented with inulin or GOS, with or without 100 μM iron sulfate. *P < 0.05, **P < 0.01, n.s. non-significant compared to control (0 mg/mL oligosaccharides) and &&&*P* < 0.001; ANOVA

We tested the effects of inulin and GOS on tumor- and inflammation-promoting *E. coli* strain NC101, which also harbors the *pks* genomic island [4, 28]. We incubated *E. coli* NC101 in the presence of the highest concentrations of both oligosaccharides (40 mg/ml) and 100 μM of iron sulfate. As shown in Fig. 3a-b, inulin and GOS supplementation did not influence the growth of *E. coli* NC101. However, oligosaccharide supplementation increased the transcript levels of *clbA* compared to control (Fig. 3c). Similar to our luciferase results with EcN, when iron was added to the medium with oligosaccharides, the expression of the *clbA* gene was reduced. As shown in Additional Fig. 2a-c, inulin supplementation, but not GOS, substantially enhanced the transcript levels of *clbB*, *clbQ*, and *clbR* genes, an effect that was completely inhibited by the addition of iron to the medium.

These results indicate that inulin and GOS consistently increase the expression of the *clbA* gene in the *E. coli* strain NC101 and that this increase can be inhibited by iron supplementation.

### Oligosaccharides increase colibactin-induced cytotoxicity and DNA double-strand breaks in Caco-2 cells

**Fig. 4:**
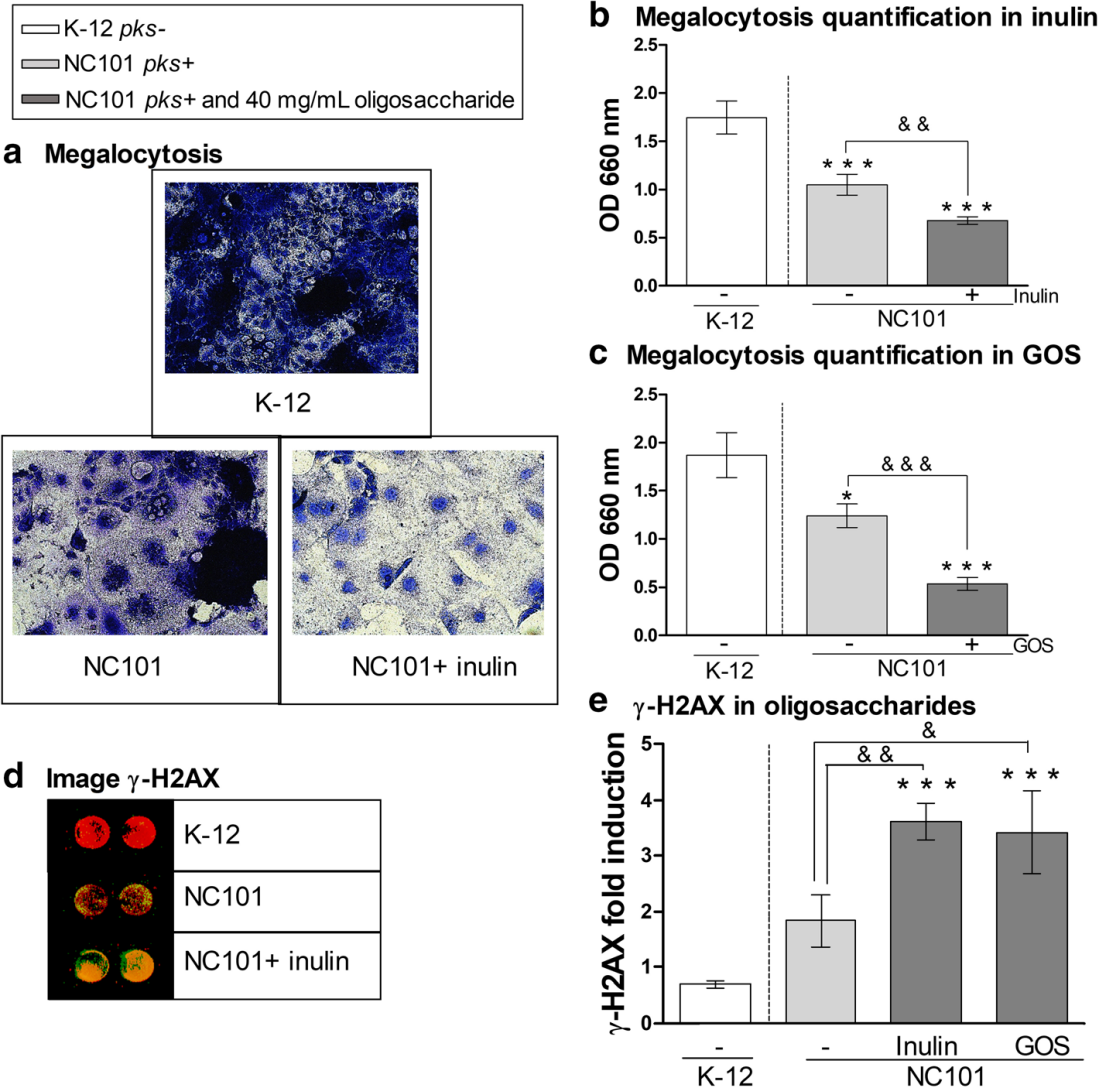
Inulin and GOS enhance DNA damage in Caco-2 cells infected with *pks+ E. coli* strain NC101. **a** Representative images of methylene blue staining for quantification of megalocytosis of Caco-2 cells 4 h post-infection (multiplicity of infection = 50 bacteria/cell; magnification 200x). *E. coli* K-12 was used as a *pks-* control strain. **b**-**c** Quantification of megalocytosis of Caco-2 cells with addition of inulin (**b**) and GOS (**c**) in medium. **d** Scan of In Cell Western image showing DNA DSBs with merged detection of total DNA (red, 680 nm) and γ-H2AX (green, 800 nm). **e** γ-H2AX fold induction in inulin and GOS (analysis were done on single colored picture). *P < 0.05, ***P < 0.001, compared to the control, *pks-* *E. coli* K-12 and &*P* < 0.05, &&*P* < 0.01, &&&*P* < 0.001; ANOVA

Our results indicate that inulin and GOS upregulate *clbA* expression and thus expression of the colibactin operon. We investigated whether this increased expression of the colibactin gene cluster at the transcriptional level could also result in greater genotoxicity. We used the *E. coli* K-12 strain, which is *psk-*, and the *pks+* strain NC101 to infect the adenocarcinoma cell line Caco-2 and assessed cytotoxicity through cell viability and the megalocytosis assay. As reported by others [24, 29], Caco-2 cell viability was reduced when infected with *E. coli* NC101 compared to *E. coli* K-12 control strain. However, a similar cell viability loss was seen in cells infected with *E. coli* NC101 in the absence of oligosaccharides and those supplemented with inulin or GOS (Additional Fig. 3a). As expected [29], Fig. 4a shows that cells infected with *E. coli* NC101 displayed enhanced megalocytosis compared to cells infected with the control *E. coli* K-12 strain. The addition of 40 mg/mL of oligosaccharides to the medium resulted in a significant increase in abnormal cell enlargement (Fig. 4a) as determined by the lower absorbance (660 nm), as shown in Fig. 4b (inulin, 1.6-fold decrease) and 4C (GOS, 2.3-fold decrease compared to cells infected with *E. coli* NC101 in the absence of oligosaccharides). Hence, most surviving cells in the inulin or GOS treated wells were megalocytic compared to untreated wells.

Taking into account the well-described effects of colibactin causing DNA DSBs [9] we used an in-cell Western assay to quantify DNA DSBs in Caco-2 cells infected with *E. coli* strains K-12 and NC101. DSBs in Caco-2 cells infected with *E. coli* NC101 were evident compared to the K-12 strain (Fig. 4d). Most importantly, the addition of 40 mg/mL of inulin led to an increase of the DSBs in Caco-2 cells as indicated by levels of γ-H2AX, a marker of DNA damage (Fig. 4e, 2-fold increase). Similar results were obtained when using GOS treatment (Fig. 4e, 1.9-fold increase).

Taken together, these data show that oligosaccharides not only increase the expression of colibactin genes, but also lead to an increase in cytotoxicity and DNA DSBs in Caco-2 cells.

## Discussion

The aim of this study was to investigate whether prebiotics, inulin and GOS, could modulate the expression and toxicity of the genotoxin colibactin, which has been linked to CRC development. Given the well-established link between diet [30], gut microbiota [31], and CRC a better understanding of how colibactin expression is regulated by different nutrients and supplements is of paramount importance due to the increasing presence of *pks+ E. coli* in healthy individuals in Western countries [5, 32] and, more recently, among Malaysian [33], Indian [34], and Japanese populations [35].

We show that fermentable fibers, such as inulin and GOS, promote the growth of colibactin-producing *E. coli* strains and elevate the expression of colibactin-related genes, as exemplified by *clbA* transcription. The *clbA* gene located in the *pks* island encodes a 4′-phosphopantetheinyl transferase and is required for colibactin synthesis [9]. Accordingly, previous studies have demonstrated that *clbA* inactivation prevents DNA damage and chromosomal abnormalities [36, 37]. Most importantly, *clbA* has been shown to contribute to the production of siderophores, which are small iron chelating compounds produced by bacteria [26]. Addition of ferric chloride to the medium inhibited the expression of colibactin related-genes [20, 27]. We further showed that addition of iron abolished the increased transcription of *clbA* induced by inulin and GOS, counteracting the effect of these fermentable fibers. This may be of interest in the context of CRC, as many patients develop anemia and are prescribed oral iron supplementation, mostly ferrous sulphate tablets [38]. Interestingly, elevated iron levels in the colon seem to be required for the probiotic activity of EcN in mice with dextran sodium sulfate (DSS)-induced colitis [39], which would have the added benefit of inhibiting colibactin expression and reducing potential EcN genotoxicity.

Consistent with increased bacterial growth and transcription of colibactin genes, inulin and GOS exacerbated the DNA damage that was induced in the *pks+ E. coli* NC101 strain. An increased number of DSBs caused by colibactin would result in the accumulation of mutations in colonic cells [40], which appear in early adenomas and accumulate in late carcinoma stages [41]. Hence, increased incidence of DSBs may accelerate the development and aggressiveness of the tumors. In fact, *pks+ E. coli* isolated from CRC patients were found to establish persistent colonization, exacerbate inflammation, and trigger carcinogenesis in mice [42].

Our findings add to the efforts in identifying environmental factors that may influence colibactin expression. For example, cinnamon and cinnamaldehyde have been shown to inhibit the expression of the *clbB* gene among several *E. coli* isolates from CRC patients [43]. More recently, a new study identified compounds of tannin and quercetin from medicinal plant extracts of *Terminalia catappa*, *Psidium guajava* and *Sandoricum koetjape* that inhibited the growth and transcription of colibactin genes of colibactin-producing *E. coli* [44].

Colibactin-related genes can also be upregulated by environmental factors, which was shown with natural food contaminants such as mycotoxins [45]. Deoxynivalenol, produced by *Fusarium graminearum* and *F. culmorum*, has been shown to exacerbate the intestinal DNA damage induced by colibactin-producing *E. coli* strains [46].

Our results are somehow unexpected, because prebiotics have been shown to have beneficial properties towards the gut microbial community. Bacterial cultures from stool samples supplemented with inulin showed a decrease in the *Enterobacteriaceae* family while promoting the growth of beneficial bacteria such as *Lactobacillus* and *Bifidobacteria* [47], and GOS was found to diminish the adhesion capacity of pathogenic *E. coli* [19]. However, those studies did not assess the potential presence and/or expression levels of colibactin genes. Furthermore, several studies in CRC rodents models showed inconsistent effects of dietary oligosaccharide supplementation on tumor growth [48]. Indeed, supplementation with 10% inulin in *Apc*^Min/+^ mice induced an increase of polyps and tumors size in the small intestine [49, 50]. However, supplementation with 15% of inulin in syngeneic wild type mice transplanted with tumors cells subcutaneously presented a slower tumor growth rate [51], and supplementation with 10% GOS in rats receiving azoxymethane and DSS to induce CRC inhibited tumor growth in the colon [52]. These studies did not assess the possible presence of *pks*+ *E. coli* strains, which may modify responses to oligosaccharides in the tested CRC models. In addition, standard rodent diets may contain varying amounts of iron between studies and, at the present, it is not clear whether dietary iron content may influence colibactin expression in the gut and affect outcomes. In any case, it should be understood that the mere presence of *pks*+ harboring strains in the gut is not sufficient to induce CRC and that there are other contributing factors [53].

## Conclusion

In conclusion we showed that, in vitro, the expression of colibactin genes and genotoxicity of *E. coli* strains harboring the *pks* island is increased by inulin and GOS supplementation. In view of the increasing usage of prebiotics and their availability as over-the-counter medicines and natural products, further experiments are needed to investigate how these prebiotics may modulate tumor development and progression in animal models and in humans in the presence of *pks+ E. coli* colonization. In addition to unveiling valuable information on the impact of oligosaccharides on colorectal tumors, further studies on specific conditions where oligosaccharides supplementation may have procarcinogenic effects could lead to personalized dietary recommendations to individuals harboring *pks+*. *E. coli.*

## Supplementary Information

**Additional file 1: Table S1.** Primers. **Figure S1.** Inulin and GOS increase the transcription of *clbB-, clbQ- and clbR*-*lux*. Growth curves of *E. coli* Nissle 1917 incubated with 0, 20, 30 and 40 mg/mL of inulin (A-C) or GOS (D-F) (OD_600_; filled symbols), and relative luminescence (RLU 10^6^; opened symbols) of *clbB-lux* (A and D)*, clbQ-lux* (B and E), and *clbR-lux* (C and F). Area under the curve (AUC) of RLU/OD_600_ for inulin (G-I) and GOS (J-L). *P < 0.05, **P < 0.01, ***P < 0.001 compared to control (0 mg/mL, oligosaccharides); ANOVA. **Figure S2.** Inulin increases the transcription of *clbB, clbQ* and *clbR* in *E. coli* NC101. Fold change of mRNA **(A)** *clbB* **(B)** *clbQ* and **(C)** *clbR* normalized on 16S rRNA expression in *E. coli* NC101 grown in minimal medium supplemented with inulin or GOS, in the absence (−) or presence (+) of 100 μM ferrous sulfate. n.s. non-significant, *P < 0.05 compared to control (0 mg/mL oligosaccharides); ANOVA. **Figure S3.** Caco-2 cell viability decreases when infected with *pks+ E. coli* strain NC101. (A) Quantification of Caco-2 cells viability with addition of 40 mg/mL inulin and GOS in medium. *E. coli* K-12 was used as a *pks-* control strain. *P < 0.05, ***P < 0.001 compared to the control, *pks- E. coli* K-12; n.s. non-significant.; ANOVA.

## Data Availability

All data generated or analyzed during this study are included in this published article and its supplementary information files.

## Abbreviations

ANOVA: analysis of variance
AUC: area under the curve
CRC: colorectal cancer
EcN: *E. coli* Nissle 1917
EMEM: Eagle′s Minimal Essential Medium
DSBs: double-strand breaks
DSS: dextran sodium sulfate
FBS: fetal bovine serum
GOS: galacto-oligosaccharides
LB: lysogeny broth
M9: minimal medium
MOI: multiplicity of infection
MTT: 3-(4,5-DimethylthiaZA-2-yl)-2,5-diphenyltetrazolium bromide
OD: optical density
PBS: phosphate-buffered saline
PCR: polymerase chain reaction
*pks*: polyketide synthetase
RLU: relative light units
rpm: revolution per minute
