# Supplementary material for: Oligosaccharides increase the genotoxic effect of colibactin produced by pks+ Escherichia coli strains
Source: BMC Cancer. 2021 Feb 17;21:172. doi: 10.1186/s12885-021-07876-8 (PMC7890614; doi:10.1186/s12885-021-07876-8)
Supplement: Supplementary file 1 — Additional file 1: Table S1. Primers. Figure S1. Inulin and GOS increase the transcription of clbB-, clbQ- and clbR-lux. Growth curves of E. coli Nissle 1917 incubated with 0, 20, 30 and 40 mg/mL of inulin (A-C) or GOS (D-F) (OD600; filled symbols), and relative luminescence (RLU 106; opened symbols) of clbB-lux (A and D), clbQ-lux (B and E), and clbR-lux (C and F). Area under the curve (AUC) of RLU/OD600 for inulin (G-I) and GOS (J-L). *P < 0.05, **P < 0.01, ***P < 0.001 compared to control (0 mg/mL, oligosaccharides); ANOVA. Figure S2. Inulin increases the transcription of clbB, clbQ and clbR in E. coli NC101. Fold change of mRNA (A) clbB (B) clbQ and (C) clbR normalized on 16S rRNA expression in E. coli NC101 grown in minimal medium supplemented with inulin or GOS, in the absence (−) or presence (+) of 100 μM ferrous sulfate. n.s. non-significant, *P < 0.05 compared to control (0 mg/mL oligosaccharides); ANOVA. Figure S3. Caco-2 cell viability decreases when infected with pks+ E. coli strain NC101. (A) Quantification of Caco-2 cells viability with addition of 40 mg/mL inulin and GOS in medium. E. coli K-12 was used as a pks- control strain. *P < 0.05, ***P < 0.001 compared to the control, pks- E. coli K-12; n.s. non-significant.; ANOVA. [file 12885_2021_7876_MOESM1_ESM.zip › Additional file 1 Oliero 2020 BMC cancer R1R2.docx]

| name | gene | sequence | reference |
| --- | --- | --- | --- |
| RT-4711up | *clbA* | CTCCACAGGAAGCTACTAAC | Homburg S. et *al.* |
| RT-4711lp | *clbA* | CGTGGTGATAAAGTTGGGAC | Homburg S. et *al.* |
| ClbB-i47024F | *clbB* | TGTTGGTGGCAACACTTGAT | This study |
| ClbB-i47129R | *clbB* | CACCTGTTCCGTTTTGTGTG | This study |
| RT-1943-up | *clbQ* | GTGAAACACTACACAGGTGG | Homburg S. et *al.* |
| RT-1943-lp | *clbQ* | CACGATCGGACAGGTTAATG | Homburg S. et *al.* |
| RT-1960-up | *clbR* | CCGTTATCTCTGCGTGAAAG | Homburg S. et al. |
| RT-1960-lp | *clbR* | AGCGTGATTCGTATTCCGAG | Homburg S. et *al.* |
| 16s1114F | 16Sl | CGGCAACGAGCGCACCCC | Lane D.J. et *al*. |
| 16s1275R | 16S | CCATTGTAGCACGTGTGTAGC | Lane D.J. et *al*. |

**Additional Table 1. Primers**

**
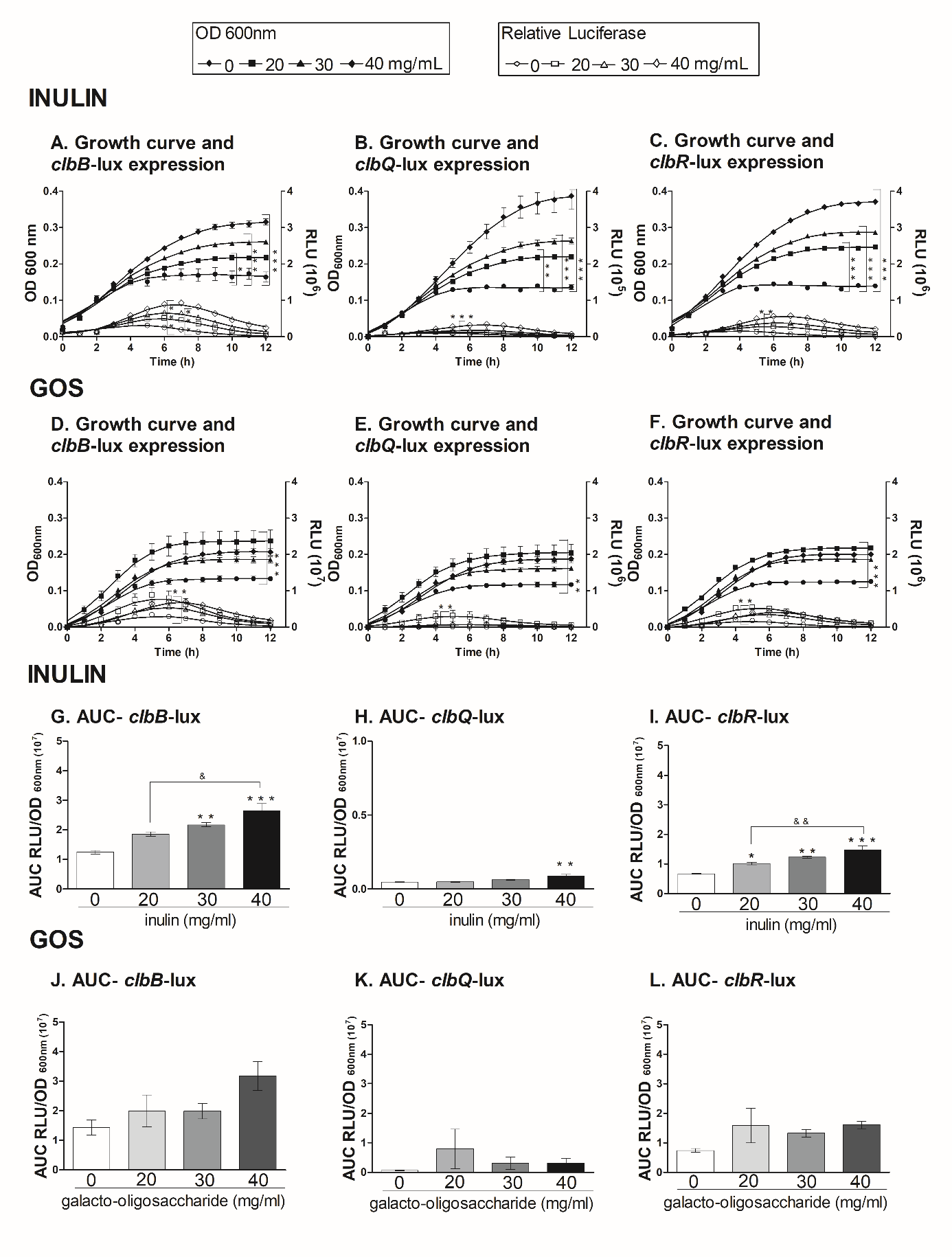
**

**Additional Figure 1. Inulin and GOS increase the transcription of *clbB-, clbQ- and clbR*-*lux*.** Growth curves of *E. coli* Nissle 1917 incubated with 0, 20, 30 and 40 mg/mL of inulin **(A-C)** or GOS **(D-F)** (OD_600_; filled symbols), and relative luminescence (RLU 10^6^; opened symbols) of *clbB-lux* (A and D)*, clbQ-lux* (B and E), and *clbR-lux* (C and F). Area under the curve (AUC) of RLU/OD_600_ for inulin **(G-I)** and GOS **(J-L)**. **P* <0.05, ***P* <0.01, ****P* <0.001 compared to control (0 mg/mL, oligosaccharides); ANOVA.


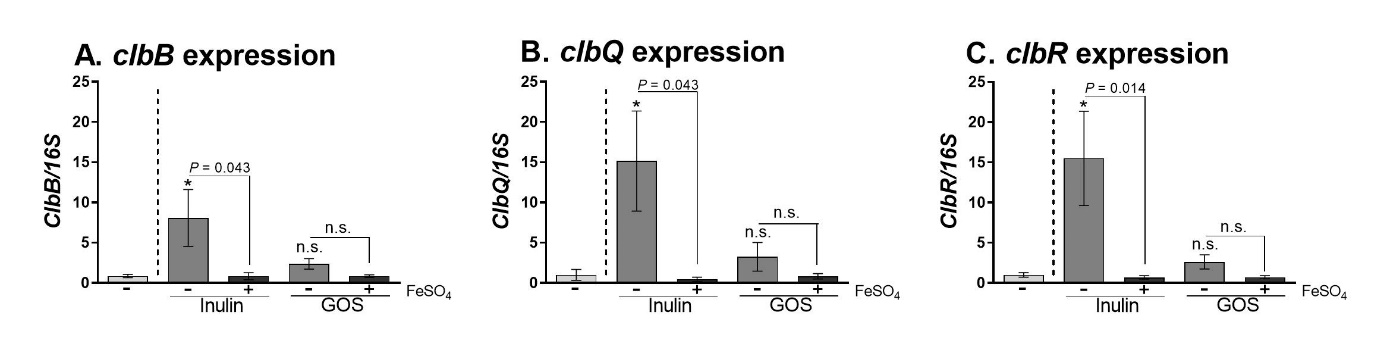


**Additional Figure 2. Inulin increases the transcription of *clbB-, clbQ- and clbR* in** ***E. coli* NC101.** Fold change of mRNA **(A)** *clbB* **(B)** *clbQ* and **(C)** *clbR* normalized on *16S rRNA* expression in *E. coli* NC101 grown in minimal medium supplemented with inulin or GOS, in the absence (-) or presence (+) of 100 µM iron sulfate. n.s. non-significant, **P* < 0.05 compared to control (0 mg/mL oligosaccharides); ANOVA.


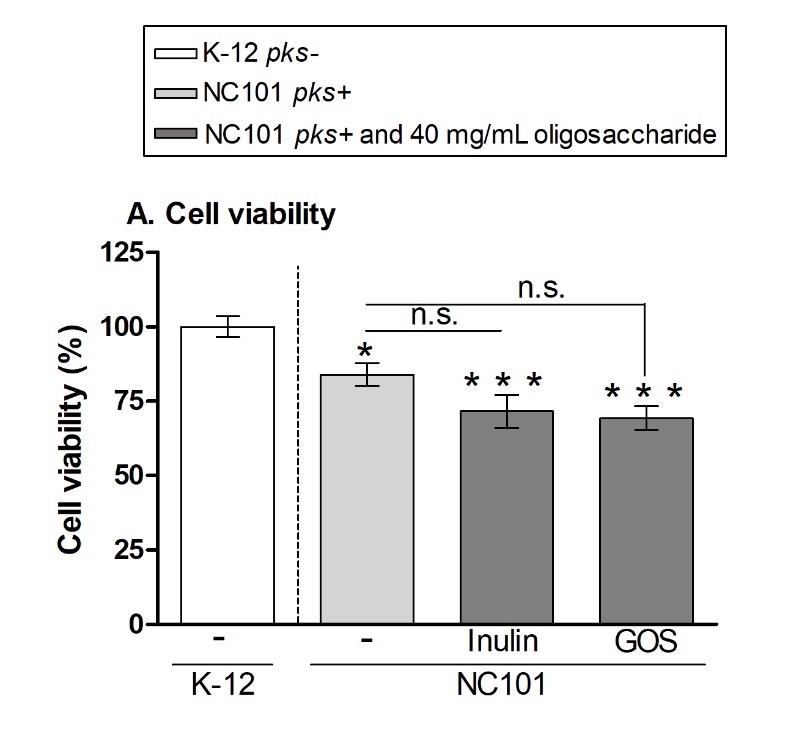


**Additional Figure 3. Caco-2 cell viability decreases when infected with *pks+* *E. coli* strain NC101.** **(A)** Quantification of Caco-2 cells viability with addition of 40 mg/mL inulin and GOS in medium. *E. coli* K-12 was used as a *pks-* negative control strain. **P* < 0.05, ****P* < 0.001 compared to the control, *pks-* *E. coli* K-12; n.s. non-significant.; ANOVA.
